# Syndrome de moelle bas attachée

**DOI:** 10.11604/pamj.2015.22.344.7615

**Published:** 2015-12-10

**Authors:** Mohamed Badaoui, Abdennasser El Kharras

**Affiliations:** 1 Service de Medecine Interne, 1^er^ Centre Médico-Chirurgical, Agadir, Maroc; 2 Service d'Imagerie Médicale, 1^er^Centre Médico-Chirurgical, Agadir, Maroc

**Keywords:** Syndrome de moelle bas attachée, dysraphisme, IRM, Tethered cord syndrome, dysraphism, MRI

## Image en medicine

Le syndrome de la moelle bas attachée est une complication du dysraphisme spinal. Affection est souvent découverte chez l'enfant, peut être asymptomatique et rencontrée chez l'adulte. Nous rapportons le cas d'une jeune fille de 20 ans qui consultait pour une symptomatologie urinaire faite d'une dysurie et d'une impériosité mictionnelle évoluant depuis 2 mois. L’échographie rénovésicale ne montrait pas de lésion spécifique en dehors d'un résidu post mictionnel estimé à 80cc. Le bilan biologique était sans particularité. La cystomanométrie objectivait une vessie hyperactive. Une IRM du rachis lombaire a révélé un cône terminal en position basse au-dessous de L5 (A) avec un cordon médullaire de signal homogène en situation postérieure (B). Une intervention chirurgicale a permet une amélioration du retentissement urinaire. Au cours de cette affection, le niveau du cône médullaire se trouve en dessous du niveau L1-L2, et souvent associé à des malformations congénitales à type de filum épais ou de tumeurs bénignes. Les examens urodynamiques ont une place dans le diagnostic et dans le suivi des syndromes de moelle fixée. L'IRM est l'examen de choix pour confirmer le diagnostic et établir un bilan complet et précis. Le traitement est essentiellement chirurgical.

**Figure 1:**
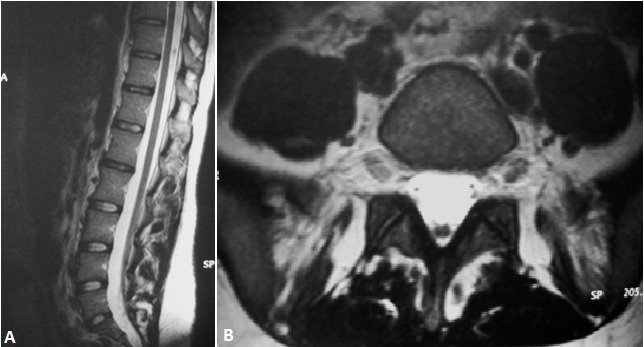
IRM lombaire en coupe sagittale (A) note une moelle bas insérée et la coupe axiale à l’étage L5-S1; (B) démontre un cordon médullaire de situation postérieure sans lésion osseuse ni des parties molles para rachidienne

